# Janus-faced citrate in aging and metabolism

**DOI:** 10.18632/aging.204138

**Published:** 2022-06-17

**Authors:** Wei-Sheng Lin, Pei-Yu Wang

**Affiliations:** 1 Department of Pediatrics, Taipei Veterans General Hospital, Taipei, Taiwan; 2 School of Medicine, National Yang Ming Chiao Tung University, Taipei, Taiwan; 3 Graduate Institute of Brain and Mind Sciences, College of Medicine, National Taiwan University, Taipei, Taiwan

**Keywords:** ketogenesis, insulin, citrate transporter

Citrate, one of the tricarboxylic acid cycle intermediates, plays an important role in cellular energy metabolism. Citrate is also a common food ingredient, occurring in numerous juices and beverages, and potassium citrate has been used to treat kidney stones in some patients. However, the long-term effects of exogenous citrate administration on overall health has not been well explored. In a recent study, we demonstrated that dietary citrate supplementation was associated with lifespan extension, decreased hemolymph glucose and triglyceride, and reduced ATP/ADP ratio in fruit flies fed on a relatively high-calorie diet [1]. Furthermore, we found that AMP-activated protein kinase (AMPK) was activated and target of rapamycin (TOR) signaling was suppressed in these flies. In line with the aforementioned findings, parallel experiments in mice fed a high-fat diet showed similar metabolic benefits for citrate supplementation, including improved glucose homeostasis and reduced hepatic lipid accumulation. These mice also exhibited better social memory and novel object recognition memory in a citrate dose-dependent manner.

Our findings imply that citrate supplementation may lead to systemic benefits that mimic the effects of dietary restriction (DR). We observed that enhanced ketogenesis was a common feature of citrate- and DR-treated animals, which suggested that the beneficial effects of citrate supplementation could be mediated by ketone bodies [2–4]. Indeed, administration of β-hydroxybutyrate, one of the major ketone bodies, extended the lifespan of fruit flies, and improved glucose homeostasis and memory performance in mice [1]. Collectively, these results suggest that citrate supplementation induces a metabolic switch toward enhanced ketogenesis under high calorie diets, which appears to be conserved across species.

It is intriguing to note that fruit flies and mice with decreased expression of a plasma membrane citrate transporter gene (*Indy*/*Slc13a5*) exhibit phenotypes that recapitulate the effects of DR, including reduced lipid storage, improved glucose homeostasis and extended lifespan [5,6]. In addition, both systematic and nervous system-specific *Slc13a5* deletion resulted in enhanced memory performance of mice [3]. At first glance, it would appear to be contradictory that citrate transporter hypofunction and citrate supplementation lead to qualitatively similar physiological effects. However, the apparent paradox may possibly be explained by the fact that these different manipulations ultimately reprogram cellular metabolism in a similar fashion.

The metabolic effects of citrate supplementation may be context dependent. For instance, citrate treatment has been associated with activation of the TOR pathway, lipid accumulation, and senescence in several tumor cell lines [7]. Dietary citrate supplementation has also been shown to induce the mitochondrial unfolded protein response and lipid accumulation in *Caenorhabditis elegans* [8]. These results are in stark contrast with the findings discussed above, however in actuality, citrate and the closely-related metabolite, acetyl-CoA, lie at the crossroads between lipogenesis (carried out mostly in the cytosol) and ketogenesis (occurring mainly in mitochondria). Additional factors, such as cellular and systemic energy status, the concentration and proportion of other intermediary metabolites, and the enzymatic activities of various metabolic pathways, may determine the direction of metabolic wiring in the face of exogenous citrate supply.

In conclusion, experimental findings to date clearly illustrate the potential translational relevance of citrate manipulation in the management of metabolic disorders, cancer, and other aging-related diseases. Further research to decipher the complexity of citrate metabolism at all levels, from cellular to organismal, is highly worth pursuing.
